# A quantitative analysis linking sea turtle mortality and plastic debris ingestion

**DOI:** 10.1038/s41598-018-30038-z

**Published:** 2018-09-13

**Authors:** Chris Wilcox, Melody Puckridge, Qamar A Schuyler, Kathy Townsend, Britta Denise Hardesty

**Affiliations:** 1 Oceans and Atmosphere, Commonwealth Scientific and Industrial Research Organisation, Hobart, Tasmania Australia; 2 Moreton Bay Research Station, The University of Queensland, Dunwich, Queensland Australia; 3 School of Biological Sciences, The University of Queensland, St Lucia, Queensland Australia; 4 School of Science and Engineering, University of the Sunshine Coast, Hervey Bay, Queensland Australia

## Abstract

Plastic in the marine environment is a growing environmental issue. Sea turtles are at significant risk of ingesting plastic debris at all stages of their lifecycle with potentially lethal consequences. We tested the relationship between the amount of plastic a turtle has ingested and the likelihood of death, treating animals that died of known causes unrelated to plastic ingestion as a statistical control group. We utilized two datasets; one based on necropsies of 246 sea turtles and a second using 706 records extracted from a national strandings database. Animals dying of known causes unrelated to plastic ingestion had less plastic in their gut than those that died of either indeterminate causes or due to plastic ingestion directly (e.g. via gut impaction and perforation). We found a 50% probability of mortality once an animal had 14 pieces of plastic in its gut. Our results provide the critical link between recent estimates of plastic ingestion and the population effects of this environmental threat.

## Introduction

The accumulation and persistence of plastic debris in the marine environment is of increasing concern. An estimated 4.8 to 12.7 million metric tonnes of plastic debris entered the world’s oceans from land-based sources in 2010 alone, with this input likely to increase exponentially into the future^1^. This poses a considerable threat to marine life, primarily through entanglement and ingestion^2,3^. While entanglement can have devastating effects, particularly those when it involves fishing gear^4^, ingestion of anthropogenic debris is also of increasing global concern^2,5,6^.

Sea turtles were among the first taxa recorded to ingest plastic debris^7,8^, a phenomenon that occurs in every region of the world^9^ and in all 7 marine turtle species. Globally, it is estimated that approximately 52% of all sea turtles have ingested plastic debris^3^; however, this varies considerably between regions. For example, 90% of juvenile green turtles off Brazil in the South West Atlantic^10^ and 80% of juvenile loggerheads in the Western Mediterranean^11^ had evidence of plastic ingestion, and 100% of turtles surveyed in coastal Brazil had ingested plastic^12^. Ingestion can occur at all stages of a sea turtle’s lifecycle; however, it appears to be most frequent in juvenile and pelagic stages^13^.

While ingestion may occur as a result of indiscriminate feeding and simply be a function of what sea turtles encounter in their environment, recent analyses suggest that plastic physically resembling turtles’ natural food is ingested at a higher rate than other types^14^. Additionally, the rate at which debris is ingested may increase when natural food intake is compromised or reduced^15^, suggesting a compounding effect. Debris ingestion can have a range of effects from a benign response, where items simply pass through the gastrointestinal tract, to lethal effects caused by gut impaction or perforation.

While there is clear evidence that ingestion of plastic debris is widespread, and evidence that ingestion has sub-lethal to lethal effects on turtles^2,16^, to date no quantitative relationship between the amount of plastic ingested by a turtle and the consequences for the individual has been established. In part, this is due to the difficulty of running controlled trials, as would be done in toxicology or other fields, to establish a dose-response relationship between plastic ingestion and fitness consequences. In the absence of controlled trials, coastal stranding information and necropsies of dead turtles may provide the best available information to estimate the dose-response relationship.

**Figure 1:**
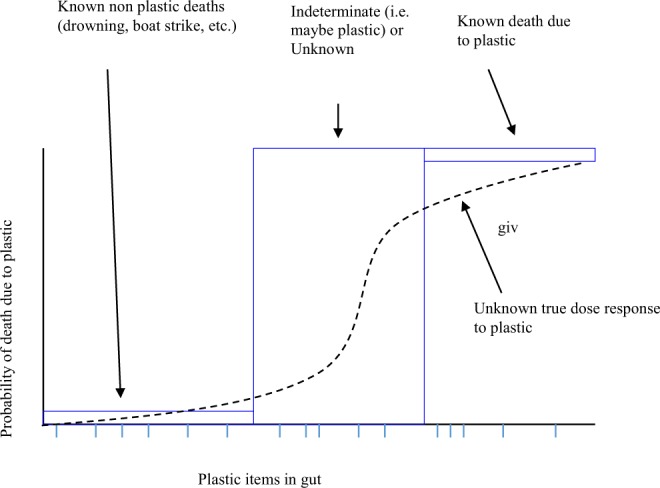
Conceptual framework for estimating the probability of death due to plastic debris ingestion. Blue lines along the x axis show observations of turtles with plastic in their gut. Blue boxes with labels show the hypothesized location along the x and y axis of the animals with various fates, if plastic causes lethal effects. The black dotted line shows a hypothesized, but unobserved, relationship between the amount of plastic in the gut and the likelihood of mortality due to plastic for an individual turtle.

Turtles in coastal environments die of many different causes, including non-plastic-related reasons such as boat strikes and entanglement in fishing nets. We used these animals as a control group, assuming their mortality was random with respect to the amount of plastic they ingested. We compared these animals to those that died of indeterminate causes, which could include plastic, and to those that were identified as dying due to plastic ingestion (e.g. gut impaction or perforation) based on necropsies. We assessed the probability of death due to plastic for sea turtles. First, we investigated whether plastic load is lowest in animals dying due to non-plastic related causes, increasing for animals potentially killed by plastic ingestion, and highest in those turtles identified as killed by plastic ingestion (Fig. 1). Then, we utilized all turtles in the dataset to estimate the relationship between the probability of death due to plastic ingestion and the concentration of plastic in the animal’s gut.

## Results

### Necropsies to assess debris ingestion

Of the 246 turtles examined, 58 (23.6%) contained debris: 13 of 24 post hatchlings (54.2%), 41 of 175 juveniles (23.4%), 2 of 13 sub-adults (15.4%), and 2 of 12 adults (16.7%). No hatchlings (22 sampled) were recorded as having ingested debris. The count and mass of debris ranged from a single piece to 329 pieces, weighing between <0.01 g to 10.41 g. Weights of debris were not recorded for 11 of the turtles. Weights <0.01 g were subsequently classed as 0.01 g to give them an absolute value for analyses.

The best model, based on Akaike Information Criterion (AIC), for the relationship between the number of debris items in the gut and the cause of death includes a main effect for age class and species (Table 1). Debris counts were significantly higher in juvenile turtles than in adult turtles, with weak evidence that post-hatchling turtles are elevated relative to adults (Table 2). Green turtles had higher levels of debris ingestion than hawksbills, while the other species did not differ from green turtles (Table 2).

**Table 1:** AIC results for models of debris in the GI tract of turtles.

Models	AICs
CAS	519.5
CA	521
CS	524.8
CA:S	525.4
C	526.4
0	588.6

Model codes are 0 – intercept only, C – cause of death, A – age class, S – species, – main effects and interaction between adjoining covariates. Note that models with main effects or interaction terms also include an intercept term. Two units of AIC implies a model is a significant improvement. Lower AIC values represent improved model fits. CAS and CA are equivalent models.

**Table 2:** Coefficient estimates for the best model (CAS) for the amount of plastic debris found in a turtle.

	Estimate	Std. Error	Pr( > |z|)
Cause Ukn	−6	1.4	0.000019*
Cause KNP	−2.5	1.1	0.027*
Cause Ind	−0.63	2.2	0.77
Cause KP	1.4	1.2	0.26
Age Class Hatchling	−32	9.5 × 10^6^	1
Age Class Juvenile	3.1	1.2	0.008*
Age Class Posthatchling	2	1.4	0.17
Species Hawksbill	−2	0.6	0.00083*
Species Loggerhead	−1.3	2	0.52
Species Olive Ridley	−34	6.7 × 10^7^	1
Species Unidentified	−33	4.1 × 10^7^	1

Cause denotes Cause of Death (CoD). Codes for causes are Ukn – unknown, KNP – known, not plastic ingestion, Ind – indeterminate, KP – known, plastic ingestion. * indicates significance at p < 0.05.

**Figure 2:**
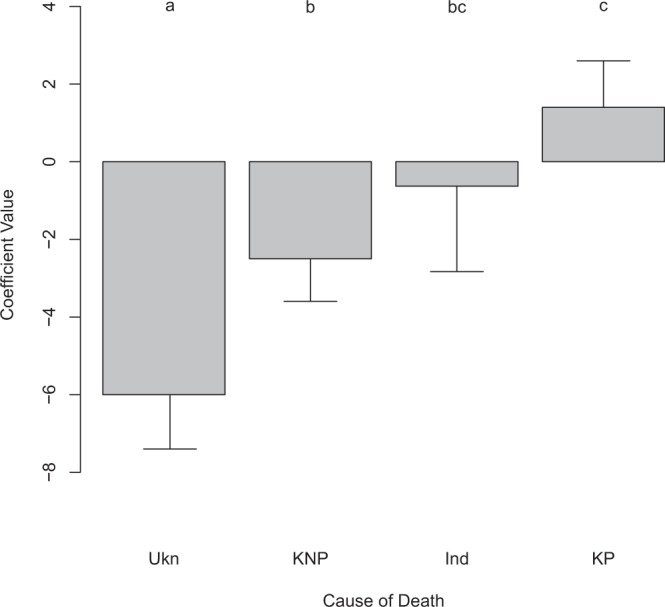
Comparison between coefficients for cause of death, from a linear regression of debris count in the GI tract on the cause of death. Codes for cause of death are: Ukn, Unknown; KNP, Known Not Plastic; Ind, Indeterminate; and KP, Known Plastic. Coefficients are from the best model, based on AIC, including cause of death, species, and age class. Error bars show standard error on the coefficients. Letters denote coefficients that are significantly different (p < 0.05) based on pairwise tests with a multiple comparison correction.

The amount of plastic debris in turtles increases according to the cause of death as follows: Unknown (non-plastic) < known (non-plastic) < Indeterminate (possibly plastic) < known (plastic), as hypothesized (Fig. 2). The unknown category was significantly lower in debris concentrations than other categories. Animals with known causes of death that were not due to plastic ingestion had significantly lower debris in their gastro intestinal tract (GIT) than those whose death was due to plastic ingestion (Fig. 2). The coefficient for animals whose cause of death was indeterminate but could have included plastic ingestion was intermediate between the non-plastic and plastic ingestion causes, but not significantly different from either.

**Figure 3:**
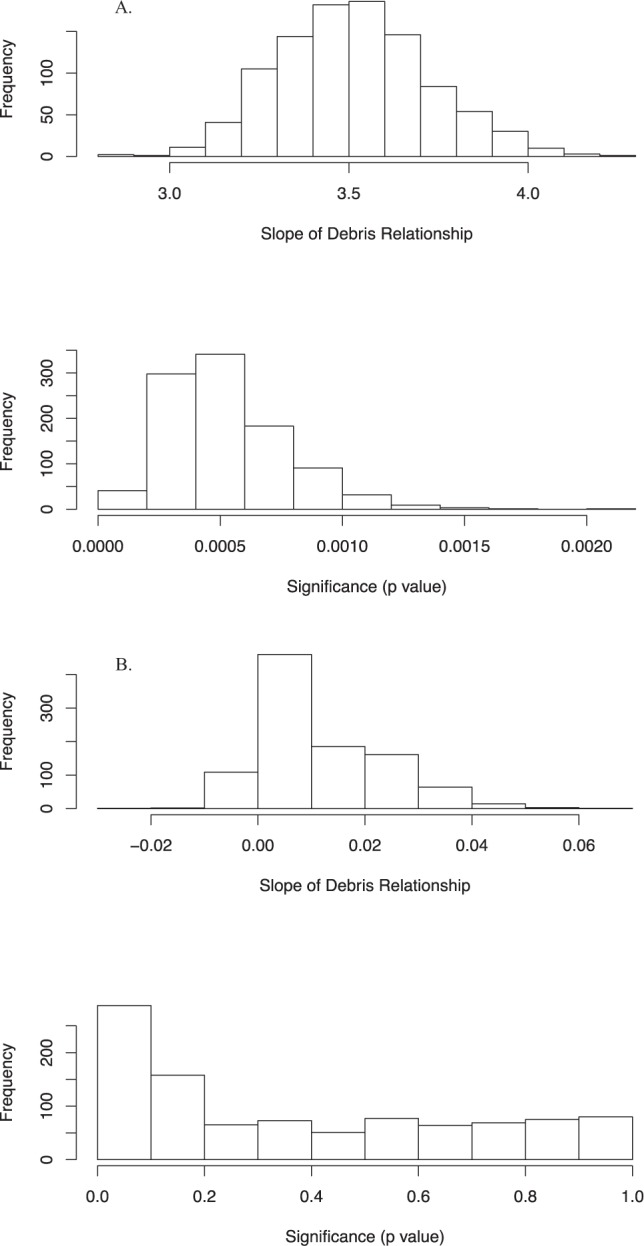
Slope and significance of the relationship between probability of death due to plastic debris and the plastic debris load in the animal. (**A**) Analysis using the cause of death as identified during necropsy. The load in this panel is the number of items per cm of curved carapace length, as per the best model in our analysis. (**B**) Analysis using a conservative form of the cause of death, where plastic ingestion related deaths are treated as indeterminate deaths. The load in this model is the number of plastic items, as per the best model for this modified data in our analysis. In each panel the top plot shows the distribution of slope estimates for the number of debris items in the gut, the lower plot shows the significance of these coefficients, from 1,000 Monte Carlo regression analysis samples.

**Figure 4:**
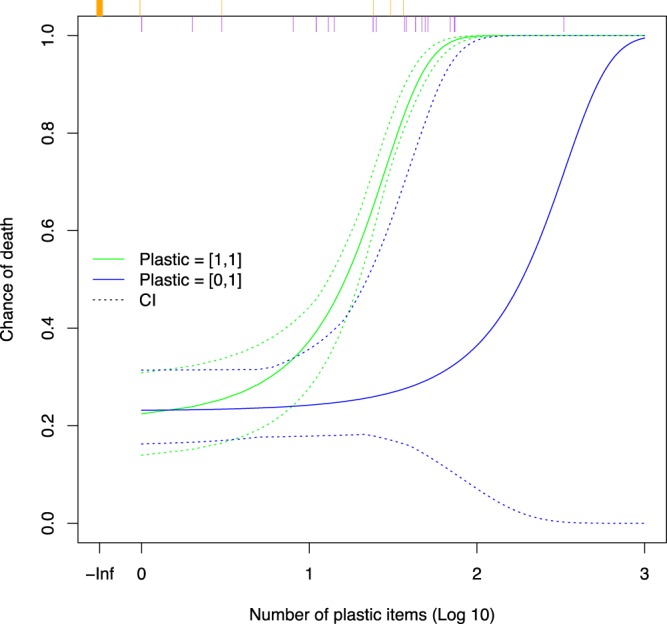
Probability of mortality due to plastic ingestion with increasing plastic load in the gastro-intestinal tract. Model results are based on the median curved carapace length for animals in the data (43.5 cm) and the most common age class (juvenile). Two models are shown, both based on Monte Carlo simulations. The first model assumes the cause of death has been assigned correctly, leading to animals with plastic ingestion as an assigned cause having a probability in the interval [1,1] in the Monte Carlo process. The second model assumes plastic has been assigned incorrectly, leading to a probability in the interval [0,1]. For each model we show the median (solid) and the extreme values (dotted) over 1,000 Monte Carlo simulations. The rug plot along the top of the figure shows the number of plastic items in each of the turtles in our samples, with orange (top) showing turtles that died of known non-plastic ingestion causes, and purple (beneath) indicating those that died of either plastic ingestion or were indeterminate.

The best model for the relationship between the number of debris items in the gut and the probability of death due to debris ingestion included the ratio of the count of items to the curved carapace length (CCL; number per centimeter) and the age class of the animal (Table 3). The coefficient for the count per centimeter has a significantly positive slope term across all of the simulations (Fig. 3). In the more conservative analysis, the simpler model with only the count of debris items and the carapace length was the best model. This is likely due to the reduced power of the data to distinguish differences among models in this case (Table 3). We used the model including count - CCL ratio and age class for predictions (Model 3a, Table 3), as the other more conservative model provides similar results, but this model includes more information likely to be of ecological relevance. Using the median values of the regression parameters from the Monte Carlo analysis, along with the median curved carapace length of animals and assuming juvenile age class (the most common) in our data, we were able to predict the relationship between the load of plastic in the gut and the probability of death due to plastic ingestion (Fig. 4). Given the mix of CCL and age classes in our data, we estimate that a load of 14 items, corresponds to a probability of mortality of 50%. Using the bounds of this relationship across all estimated values from our Monte Carlo simulations, we found a relatively small amount of uncertainty (Fig. 4). Evaluating the robustness of our results, we found that treating indeterminate and plastic causes of death similarly, and repeating the Monte Carlo analysis and subsequent predictions resulted in qualitatively similar results, although with a broader uncertainty (Fig. 4).

**Table 3:** AIC results for the alternative models for the dose response relationship between plastic load and death.

Model Version	Model Formula	AIC
1a	Count + CCL	237.7
2a	Count + CCL + NW Residuals	239.1
3a	(Count/CCL) + Age Class	236.7
1b	Count + CCL	242.5
2b	Count + CCL + NWResiduals	243
3b	(Count /CCL) + Age Class	244.2

Model version 1a to 3a assume cause of death is correctly assigned for death due to plastic ingestion. Models 1b – 3b assume that death due to plastic may be incorrectly classified in some cases, and thus these observations are treated similarly to indeterminate cases in the Monte Carlo regression analysis. Differences between nested models of 2 or more AIC units are considered statistically significant. Versions a and b are not directly comparable, as the underlying data is different due to the Monte Carlo procedure. See methods for details. In the table Count is the total number of plastic pieces in a turtle digestive tract, CCL is curved carapace length, NW Residuals are the residuals from the relationship between number of plastic pieces and total weight, and Age Class is the age class of the turtle, as explained in the methods.

### StrandNet records to assess debris ingestion

**Figure 5:**
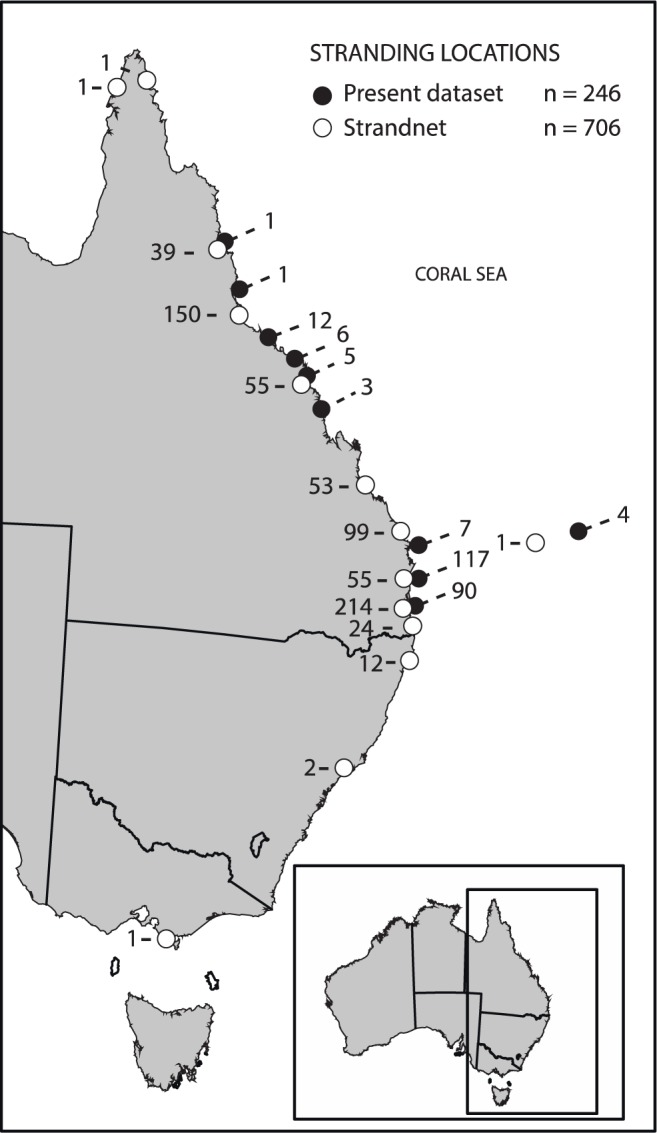
Locations of stranded turtles. Map of the Eastern seaboard of Australia (lower right) with sample locations for stranded turtles that were necropsied based on data from authors in this study (black circles) and Strandnet (white circles). Map made using ArcGIS 10.3^32^.

**Figure 6:**
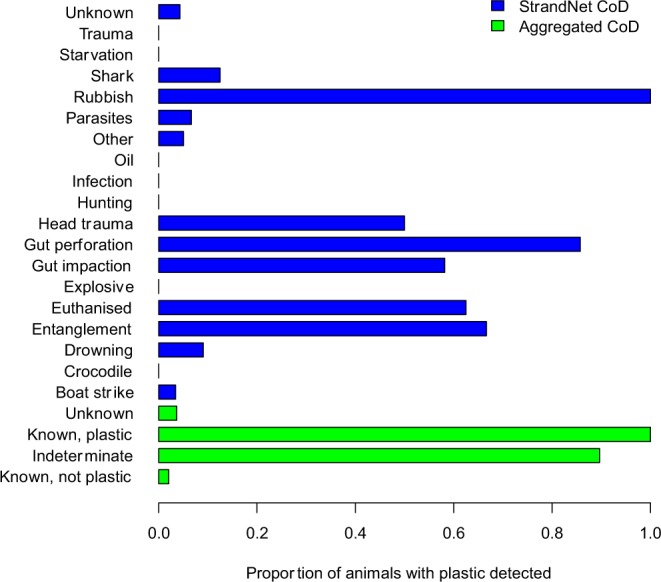
Proportion of animals with plastic debris reported in their gastro-intestinal tract for different causes of death, as reported in StrandNet reports (blue) and aggregated for analysis (green). Note that assignments to an aggregated cause of death depended on examination of each necropsy report, and thus there is not a single translation of the StrandNet cause of death to the aggregated cause.

We extracted 706 necropsy records from StrandNet (Figs 5, 6). Of these, 345, 170 and 6 had gastro-intestinal tract (GIT) contents examined to a level of 1, 2, and 3 respectively. There were an additional 185 records with no evidence the GIT contents were examined (i.e. level 0). However, plication, or tangling, of the gut from fishing line ingestion is visibly evident without opening the GIT. Therefore, 13 instances where cause of death was attributed to debris ingestion were recorded despite lack of explicit mention that the GIT was opened for inspection. There was also an additional case where a plastic bag was seen coming from the cloaca of an animal, yet no clear indication the GIT contents were examined. In this case, cause of death was scored as unknown. Due to the lower specificity of StrandNet records with respect to recording marine debris data, we scored each reported animal as debris present or debris absent. Age class was simplified to a binary classification of adult/not adult (based on carapace length) given the distribution of observations across the possible classes. Species was not included in the analysis due to low representation of several species. For example, the leatherback (*Dermochelys coriacea*), olive ridley and flatback turtles had only 4, 10 and 12 individuals represented respectively. Nonetheless, all species had ingested debris.

**Figure 7:**
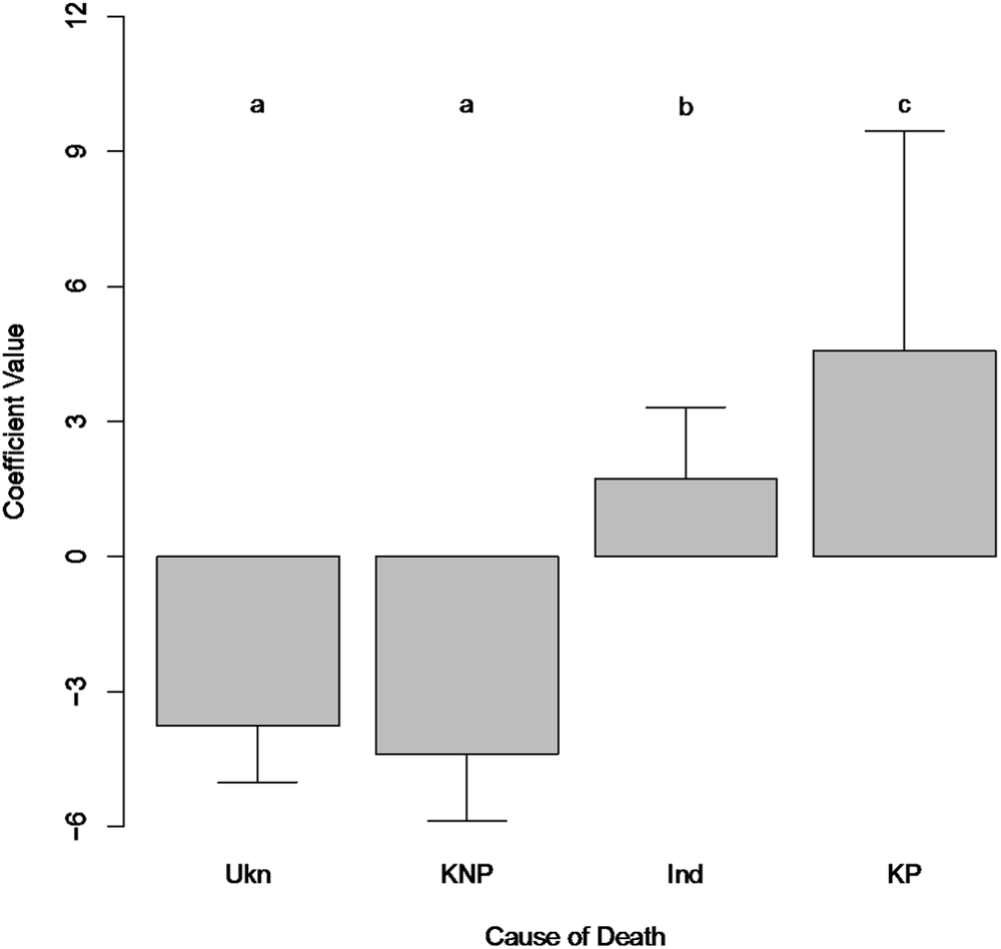
Coefficient values for the effect of the cause of death on the presence of plastic in the gastro-intestinal tract of turtles, reported in StrandNet. Codes for cause of death are: Ukn, unknown; KNP, Known Not Plastic; Ind, Indeterminate; and KP, Known Plastic. Error bars show 95% confidence intervals on the regression parameters. Letters show significant differences (p < 0.05) among parameters.

The best-fit model included the cause of death, a binary age class (adult/not adult), and a binary exam variable (level of examination > 0 vs. level = 0), with both simpler and more complex models having significantly worse AIC scores. Higher levels of examination were more likely to result in debris detection, with adult turtles having lower frequencies of debris detection than non-adults (Table 4). Debris was least likely to be found in animals that died of unknown or non-debris related causes (Table 4). Animals whose cause of death was indeterminate (but could have included debris) were more likely to have debris in their gut (Table 4). Animals with plastic ingestion as the known cause of death had the highest frequencies of plastic in their digestive system (Table 4). Pairwise comparison of the coefficients demonstrated that unknown and known non-plastic causes were not different, but were both significantly below indeterminate causes, which were significantly below known deaths due to plastic ingestion (Fig. 7).

**Table 4:** Best fitting model for the presence/absence of plastic debris in turtles reported in examination forms in the StrandNet database.

	Estimate	Std. Error	Pr( > |z|)
Cause Ukn	−3.8	0.53	0
Cause KNP	−4.4	0.67	0
Cause Ind	1.7	0.71	0.0078
Cause KP	4.6	1.4	2.00E−08
Age Class Adult	−1.2	0.64	0.058
Exam Level > 0	0.98	0.56	0.068

Cause denotes Cause of Death (CoD). Codes for causes are Ukn – unknown, KNP – known, not plastic ingestion, Ind – indeterminate, KP – known, plastic ingestion.

## Discussion

We found strong support for the two hypotheses we posited with respect to plastic ingestion based on our necropsy data. First, animals that had plastic ingestion listed as the cause of death had higher concentrations of plastic debris in their gastro-intestinal tract than animals that died of known non-plastic ingestion related causes. We found that the causes of death are segregated in terms of plastic concentration (Fig. 1). Second, when we regressed the probability of death due to plastic on the concentration of plastic in the gastro-intestinal tract, we found strong support for a positive relationship, suggesting that higher concentrations of plastic items in the gastro-intestinal tract lead to a higher probability of mortality. Analysis of the government strandings database (Strandnet) generally supported the results from our necropsy data. The proportion of animals with plastics in their gut increased as we hypothesized, with unknown and known not due to plastics < indeterminate < known due to plastics.

Our results can be used to predict the mortality probability for any turtle, given a CCL, age class and load of plastic items in the gastro-intestinal tract (see SI). For instance, for a juvenile animal with a CCL of 43.5 cm (the median size in our dataset), the probability of mortality rises to 0.5 (i.e. 50% of animals would be expected to die) with a load of 17 items. This increases to a probability of 1.0 (i.e. certain death) at 226 items. Our results also indicate that animals can die due to the effects of plastic ingestion, even when they have ingested a single item. For example, two animals had ingested only one plastic item, yet this item had caused the animal’s death. In one case this was due to a gut perforation, and in the other it was due to gut impaction. Our analysis suggests that at this lower end, there is a 22% chance of dying due to ingesting a single debris item.

The structure of the sea turtle gastro-intestinal tract and the mixture of anthropogenic debris items they ingest both play a role in the relationship between plastic ingestion and the chance of mortality. The alimentary canal in sea turtles is particularly prone to plication and accumulation of debris items due to the inability of turtles to regurgitate items and their convoluted gastrointestinal tracts. The nature and type of debris can have compounding effects in the accumulation of debris. For example, normal gut passage time for sub adult and juvenile loggerhead turtles is estimated to be between approximately five and 23 days^15,17^. However, transit time for items through the GIT is strongly influenced by the individual characteristics of the item ingested (e.g. density, size, shape)^17^. One feeding experiment found that, rather than passing through the GIT individually, pieces of soft plastic could compound together and pass as a single compacted item, despite being ingested at separate intervals^15^. Their capacity to form an obstruction is also fuelled by the extended time until elimination, noted to be up to four months for small pieces of soft latex^15^ and even up to six months for 10 × 10 cm plastic sheets^18^.

In addition to the interaction between plastic type and gut passage time, plastics themselves differ widely in their likelihood of causing perforation or impaction. For instance, a single piece of monofilament fishing line or a metal hook can cause plication as it passes through the gut^19^. In contrast, small hard plastic fragments may pass quickly through the gut with little incident. Furthermore, turtles likely differ in their exposure to amounts and types of plastic, based on their feeding location in the water column and with respect to the coastal zone. Plastic fragments on the water surface have been found to be larger near coastal zones and in the gyres, presumably due to their proximity to coastal sources or collection points in the gyres^1,20^. As such, one would expect turtles feeding in surface waters near coastal margins to be more likely to ingest larger fragments that would be more likely to cause mortality.

This interaction between feeding location, plastic characteristics, and life history stage is reflected in our data. Twenty-three percent of the juveniles and fifty-four percent of post-hatchling stage turtles in our necropsies ingested plastic, in comparison with fifteen percent of the sub-adults and sixteen percent of adults. The younger animals are feeding in the water column nearer the surface, and in some cases in coastal environments where debris is potentially bigger or in convergence zones where plastics accumulate. Thus, in using our results to translate rates of plastic ingestion into probability of mortality, it is important to consider both the life history stage of the animal and the location where it is feeding.

There have been discussions on sea turtles and debris selectivity. A study on the visual similarities between their natural prey items and the plastic debris ingested suggests sea turtles will actively seek out plastic debris that appear similar to their food sources, particularly flexible film-like items^13^. However, this does not preclude instances where ingestion occurs incidentally. Regardless, post hatchlings and juveniles are shown to have higher incident rates of ingestion^9^. These turtles occupy pelagic environments and are more likely to feed in areas where debris accumulates (surface waters and convergence zones)^9^.

Pelagic juveniles caught in longline fisheries operating in convergence zones in many parts of the world show relatively high rates of plastic ingestion^10,21^ suggesting that high volumes of debris in seemingly healthy juveniles is a widespread phenomenon. All four pelagic juvenile turtles that drowned on longlines included in our data are consistent with these findings, showing relatively high levels of debris ingestion (between 24 and 39 pieces, Table 2). Our model would suggest these animals have relatively high mortality probabilities from plastic ingestion, between 0.69 and 0.90 (Fig. 4), yet plastic ingestion was not fatal for these turtles, as the animals died from drowning.

This apparent contradiction may point to one bias in our data and analysis. Most of the animals we necropsied, and those included in StrandNet, were sourced from coastal areas where they have washed up either incapacitated or dead. These coastal animals have died from a mixture of causes, with the cause of death not strongly related to the likelihood of sampling the animal. The pelagic juveniles differ, as the cause of death is directly related to the chance of sampling an animal. Only animals caught in pelagic longlines were recovered and necropsied. This implies we have no samples of animals that have died due to plastic ingestion which would correspond to the longline caught animals. If plastic debris in offshore regions is smaller and more compactly shaped, it might be that there is a significant difference (reduction) in the likelihood of mortality due to ingestion of plastic debris in these regions. This again suggests that our model should be used primarily in coastal regions, and if applied offshore should be considered an upper bound on the probability of mortality.

Nearly 700 species are now known to interact with anthropogenic debris^22^ and as more species are investigated, the number continues to rise. As global plastic production increases, so too does our understanding of the ubiquity and impacts of anthropogenic debris on marine fauna such as seabirds^2,23^, fish^24,25^, marine mammals^2,26^, and a range of invertebrates^27,28^ including corals^29^. This work provides a critical next step in quantifying the risk plastic pollution poses to the world’s declining sea turtle populations, by linking plastic debris loads and the likelihood of mortality. The model has broad applicability and can be adapted for other taxa to understand dose responses to plastic ingestion for other marine taxa of interest.

## Methods

Two sources of data were collected for this study; the first was a necropsy dataset specifically investigating plastic ingestion throughout the entire gastrointestinal tract (GIT) and the second was a dataset that investigated plastic ingestion from necropsy reports lodged with the Queensland Government StrandNet database (https://www.derm.qld.gov.au/strandnet).

### Necropsies to investigate debris ingestion

#### Material collection

Necropsies were performed on 246 sea turtles obtained from across Queensland, Australia (Fig. 5) that had either stranded dead or were alive and subsequently died after a period of time in a rehabilitation facility. Of these, 160 were green sea turtles (*Chelonia mydas*), 52 were hawksbill turtles (*Eretmochelys imbricata*), 30 were loggerhead turtles (*Caretta caretta*), one was a flatback turtle (*Natator depressus*) and one was an olive ridley turtle (*Lepidochelys olivacea*). There were also two unidentified deceased turtles. The turtles ranged from hatchlings through to adults (CCL ranged from 4.1–116.3 cm), however, the great majority were juveniles with an overall average size of 42.0 cm CCL. Necropsies followed procedures described in Wyneken^30^. Cause of death (COD) was attributed where possible. When this was uncertain (the majority of cases), COD was simply assigned to ‘unknown’. Contents of the gastrointestinal tract were sieved to extract any plastic present. When found, plastic was classified into material type, measured, counted and weighed. Animal ethics permits were not required to carry out necropsies, as animals were deceased.

#### Data preparation

To investigate a potential dose-response relationship between debris and death, we first classified each death into four general causes: (1) unknown (Ukn), where there was no clear evidence of or cause of death, including absence or very low levels of plastic in the digestive system; (2) known, non-plastic ingestion related (KNP), where there was a clearly identifiable cause such as drowning in fishing gear; (3) indeterminate (Ind), where there was substantial plastic debris present in the digestive system but also other possible causes, such as infection or propeller cuts; and (4) known, plastic ingestion related (KP), where there was gut blockage, perforation, or other clear signs of plastic driven mortality. If plastic debris causes death, it is expected that the amount of debris present should scale as: Ukn & KNP < Ind < KP. This can be seen conceptually in Fig. 1, where the three fates occupy the blue boxes respectively in the plot, with volume of debris increasing along the x-axis.

#### Analyses

All analyses were performed using R version 3.1.1^31^. First we tested for differences in the amount of plastic debris (measured as the count of items) present in the four fate categories using a generalized linear regression model. We utilized a negative binomial error, due to over-dispersion in the data, which proved adequate based on a Chi square test. In addition to cause of death, we included age class and species as variables, as these can influence the frequency of debris ingestion^9^. We identified the best model using the Akaike Information Criterion (AIC). Once identified, we used the best model to estimate the pairwise differences between the coefficients for cause of death to determine if they were in the hypothesized order Ukn & KNP < Ind < KP (Fig. 1).

We then assigned an interval value for the probability of death due to plastic ingestion, for each animal. Animals with known causes other than plastic ingestion (KNP, e.g. boat strike) were assigned [0,0], animals with known causes due to plastic ingestion (KP) were assigned [1,1]. Animals with either unknown causes, or indeterminate causes (i.e. plastic debris is one of many factors noted) were assigned the range [0,1]. We then used a logistic regression to relate the probability of death due to plastic ingestion to the number of plastic items in an animal’s gut. In order to accommodate the interval values for the unknown and indeterminate causes of death, we used a Monte Carlo technique. We randomly drew a value in the interval [0,1] for each observation in these two categories (Unknown and Indeterminate), fit the model to the full dataset across all four causes (Unknown, Known not plastic ingestion, Indeterminate, and Known plastic ingestion), and captured the estimated coefficient for the number of plastic items in the gut and its standard error. We repeated this process 1,000 times, and report the distribution of the coefficient and its significance.

We tested three alternative models for the relationship between plastic load and probability of death due to plastic. The simplest model accounted for the effect of gut volume on the relationship between chance of death due to plastic and the number of items by including the curved carapace length of the animals as a covariate, on the assumption that it is roughly proportional to gut volume. There is a potential for animals of different age classes to be exposed to differing debris compositions. For instance, coastal adults might encounter mostly larger plastic debris that has recently washed into the ocean, while pelagic early juveniles might encounter smaller fragments that have degraded offshore. We tested two alternative models (see SI for further detail) in the Monte Carlo analysis. One model included the residuals from the relationship between the number of debris items found in a turtle and the dry weight of the items as a predictor, in addition to the total number of items. This allowed us to capture the size distribution of the items, without the issues of collinearity that would arise if we included both the number and the total weight directly. The second alternative utilized the number of items divided by the curved carapace length, as a measure of the amount of debris in a turtle standardized for its size.

We also included a factor for age class, which would allow the debris density (i.e. number of items per unit of curved carapace length) to differ in its effect for each of the age classes. For the regression analyses we aggregated the three adult subclasses into a single adult group, yielding 4 age classes: Hatchling, Post-Hatchling, Juvenile and Adult.

### Historical stranding data

#### Data collection

To determine how representative our findings are in a broader context, we analysed reports on turtles stranded between 1992 and 2014 reported to StrandNet (the Queensland state government’s stranding reporting system). Because the only way to determine if plastic was ingested is by necropsy, we restricted our analysis to include only animals that had been necropsied. The extent of data from StrandNet reported necropsies varied widely. Hence, we scored the level of examination of the GIT contents as(0) not examined or no evidence of examination^1^, basic examination; when contents of only one portion of the GIT were stated^2^, examined; when contents of > 1 region of the GIT was stated and^3^ comprehensively examined; when GIT contents were sieved similar to the present study.

Plastic densities were then classed as (a) Present, (b) Not present or (c) None noted; as many cases made no explicit mention of plastic debris presence or absence. When available, debris characteristics (numbers and debris type) were included. We also recorded the cause of death, as reported in the StrandNet record. Just as in the necropsy analysis we aggregated the causes of death in Strandnet to four categories, (1) cause of death unknown (but with no or very low levels of plastic in the digestive system); (2) known and not related to plastic ingestion; (3) indeterminate (plastic was present in the gut but not clearly the sole cause of death); and (4) plastic ingestion resulted in mortality.

#### Statistical Analyses

We used logistic regression to estimate the probability that debris was found in an animal conditional on its assigned cause of death, the level of examination done, age class, and species.

## Electronic supplementary material

Supplementary information
