# Supplementary material for: A quantitative analysis linking sea turtle mortality and plastic debris ingestion
Source: Sci Rep. 2018 Sep 13;8:12536. doi: 10.1038/s41598-018-30038-z (PMC6137038; doi:10.1038/s41598-018-30038-z)
Supplement: Supplementary file 1 — Supplementary information [file 41598_2018_30038_MOESM1_ESM.docx]

The regression model can be used to predict the mortality for any given turtle, using its plastic load, curved carapace length (CCL) and age class. This is done using the inverse of the logit function, which is the link in the generalized linear model that we used for the analysis of the mortality probabilities. The regression coefficients are represented by $\beta_{i}$in the following equation:

$\Pr\left( mortality|CCL,AgeClass,PlasticCount \right)=\frac{e^{(\beta_{0}+\beta_{C/ccl}\frac{count}{CCL}+\beta_{AgeClass})}}{1+ e^{({\beta_{0}+\beta}_{C/ccl}\frac{count}{CCL}+\beta_{AgeClass})}}$.

Here $\beta_{0}$ is the intercept term, $\beta_{C/ccl}$ is the term for the count of plastic items divided by the CCL in centimeters, and $\beta_{AgeClass}$ is the coefficient for the age class of the animal, as defined in the methods. For a turtle with a CCL of 43.5, age class of juvenile and a plastic load of 17 items, we substitute in the appropriate values and coefficients:

$\Pr\left( mortality|CCL,AgeClass,PlasticCount \right)=\frac{e^{(-1.048424+3.515602\frac{17}{43.5}-0.250278)}}{1+ e^{(-1.048424+3.515602\frac{17}{43.5}-0.250278)}}$.

After manipulation, this yields

$\Pr\left( mortality|CCL,AgeClass,PlasticCount \right)=0.50$.

This equation can be solved for any unknown given the regression parameters. Thus, we can calculate the load at which we have a given mortality probability, or the effect of differences in CCL on mortality at a given load, or the effect of different loads for a given CCL.

Using this model with age classes other than the juveniles, as in the equation above, requires a change in the value of the age class coefficient. The coefficients are: Hatchling – 0.2864325, Post Hatchling – 0.6206501, Adult – 0. Note that in making predictions using these equations, they are based on turtles sampled primarily from coastal Australia. Thus the plastic debris sampled is likely representative of coastal waters. Other locations with significantly different plastic waste distributions, such as in in the gyres where plastic debris tends to be much smaller, could be less accurately represented. We have also not provided the full range of parameter values for brevity, thus the calculation above is a point estimate and does not incorporate the uncertainty in the parameters.
